# The awareness of sharenting in Italy: a pilot study

**DOI:** 10.1186/s13052-024-01797-5

**Published:** 2024-10-29

**Authors:** Pietro Ferrara, Ignazio Cammisa, Margherita Zona, Elena Cimaroli, Roberto Sacco, Ivana Pacucci, Maria Teresa Grimaldi, Francesca Scaltrito, Massimo Pettoello-Mantovani, Giovanni Corsello

**Affiliations:** 1 Department of Medicine and Surgery, University Campus Bio-Medico, Rome, Italy; 2 Operative Research Unit of Pediatrics, Fondazione Policlinico Universitario Campus Bio- Medico, Rome, Italy; 3 Italian Academy of Pediatrics, Milan, Italy; 4 Institute of Pediatrics, Catholic University, Rome, Italy; 5 Child Neuropsychiatric, University Campus Bio-Medico, Rome, Italy; 6 Department of Medical and Surgical Sciences, University of Foggia, Via Pinto, 1, Foggia, 71100 Italy; 7 European Paediatric Association, Union of National European Pediatric Societies and Associations, Berlin, Germany; 8 Mother and Child Department, University of Palermo, Palermo, Italy

**Keywords:** Sharenting, Children, Neglect, Abuse, Privacy, Digital identity

## Abstract

**Background:**

This pilot study examines the widespread phenomenon of “sharenting” and how it is perceived by parents. Given the increasing prevalence of this practice, the study aims to explore parental awareness of its potential risks and assess whether it is perceived as a form of child abuse, particularly regarding the violation of children’s privacy. While traditional forms of child abuse inflict direct harm on the child, sharenting can damage the child’s image and pose risks to their well-being, both in the present and the future. We evaluated the potential correlation between specific demographic characteristics and parents’ social media usage. The study aimed to assess the possibility of expanding the research by involving European pediatric societies to obtain comparable data and develop family education programs with the goal of limiting this phenomenon. It also emphasizes the important role that pediatricians and schools can play in these programs.

**Methods:**

The study was designed using a survey model, one of the recognized quantitative research methods described in the literature and was conducted by the Pediatric Department of the University Hospital Campus Bio-Medico in Rome between November 2023 and January 2024.

**Results:**

Data from this study indicate that gender, age, education level, number of children, and number of social media accounts were not associated with sharenting phenomenon and suggest that the frequency and the onset time of sharenting were the main influencing variables in the perception of sharenting phenomenon as neglect and abuse.

**Conclusions:**

Preventive interventions, such as counseling and parental education, are essential to safeguard children’s well-being and prioritize their best interests, including their privacy and identity. Moreover, while the data from this study are limited, they underscore the importance of expanding data collection efforts across different countries. Establishing a comprehensive database could be beneficial for local and European governments in developing policies and educational programs aimed at mitigating potential risks associated with the improper exploitation of personal data, thus safeguarding children in both the short and long term.

## Introduction

The term “sharenting,” a blend of “sharing” and “parenting,” refers to the growing trend of parents sharing photos, videos, and other personal information about their children on social media profiles [1]. While the prevalence of this phenomenon varies across different studies, it often reaches significant levels, suggesting that sharenting has nearly become a social norm and a common parenting practice, frequently without the explicit consent of the children [1]. Sharenting occurs at every stage of childhood, from pregnancy to adolescence, though parents tend to post less information online as their children grow older [2, 3]. Nowadays, parents create a digital footprint for their children before they can decide whether to have an online presence: according to the results of the AVG survey, conducted in many countries including Italy, about 81% of children under the age of 2 are already involved [4–6]. Several underlying psychosocial causes have been proposed to explain the phenomenon of sharenting. Firstly, it serves as a valid tool to maintain social relationships and communicate with peers by involving friends and family members in the child’s growth and development, and to collect memories, including pictures of milestones, family and friends, and those perceived as cute or funny are the most shared on social media [4, 7]. Secondly, social media are generally used by parents to feel part of a group, enabling them to share parenting issues and problems (such as sleep, feeding, education, school, and health) and seek social and emotional support, receiving feedback from other parents [1]. The development of a self-image as a good parent and the potential for social comparison are other possible explanations for sharenting, as it can fulfill parents’ needs for self-actualization and social recognition [8, 9]. This practice can expose children, even involuntarily, to risks including privacy violation, identity theft due to the disclosure of private information, bullying by peers, possession and misuse of photos by strangers, including for sexual purposes [3]. Digital abduction is another problem associated with sharenting, where strangers take photos of children and repost them online as if the child were their own, with a new name, story, and online life. Furthermore, the child’s created digital footprint is available for social media providers and marketers who collect information from online users as valuable material for data mining or commercial purposes [5, 9]. Moreover, the caregiver may prioritize the desire to create engaging content for their followers over the needs of the child. In some cases, the caregiver may even coerce the child into repeating certain behaviors or phrases on camera to exploit the child for their entertainment’s economic value. From this perspective, sharenting can be considered a form of childhood neglect and abuse [10–13]. The aim of this pilot study was to analyze the widespread phenomenon of sharenting and parents’ perceptions of it as a form of child abuse and neglect, while evaluating the potential correlation between specific demographic characteristics and parents’ social media usage. The study also sought to explore the possibility of expanding the research by involving European pediatric societies to obtain comparable data and develop family education programs, supported by pediatricians and schools, aimed at limiting this phenomenon.

## Materials and methods

The study was designed using a survey model, one of the recognized quantitative research methods described in the literature and was conducted by the Pediatric Department of the University Hospital Campus Bio-Medico in Rome between November 2023 and January 2024. The parental version of the validated survey module developed by Ayten Doğan Keskin et al., as part of the study “Sharenting Syndrome: An Appropriate Use of Social Media?” published in PubMed in May 2023 [13] was used. The sample for the study was collected through social network sites using the snowball sampling method. To ensure sample validity, the research link was distributed to a total of 700 participants. The survey questions were administered via Google Forms. Participants provided their full consent to participate in the study. Recruitment was primarily through word of mouth and social networks. The research link was shared on various social media platforms (Facebook, Instagram, WhatsApp), and data collection commenced. During data collection, participants who completed the questionnaire were encouraged to share the survey link with other parents of children aged 0–16 years. Data collection concluded once the sample size reached a number of participants considered sufficient by the standard study protocol used for this research [13]. No participants were excluded from the sample. Following completion of the survey questions, the collected data were compiled. Percentages and frequencies of the collected data were calculated and reported. Additionally, participants’ opinions were documented through direct quotes. The relationship between variables and sharenting phenomenon was assessed using the chi-square independence test (χ²), a hypothesis test in statistics that utilizes the chi-square distribution to determine whether the null hypothesis should be rejected with 95% confidence (*p* ≤ 0.05). Multivariate analysis of categorical variables was conducted using binary logistic regression analysis. Variables influencing sharenting phenomenon were analyzed using binary logistic regression analysis. Statistical analysis was performed using IBM SPSS Statistics 24.0 software (IBM Corporation, Armonk, NY, USA). This study adhered to the regulatory standards of Good Clinical Practice and the Declaration of Helsinki (1996) and received approval from the ethical committee for the Pediatric Unit research of Campus Bio-Medico University Hospital.

## Results

A total of 405 parents with children aged between 0 and 16 years participated in the study, including 331 mothers (81.7%) and 74 fathers (18.3%). Regarding age distribution, 2% of parents were between 18 and 25 years old, 26.4% were between 26 and 35 years old, 25.9% were between 36 and 45 years old, and 45.7% were over 45 years old. In terms of education level, nearly all participants had at least a high school diploma, with only 10.9% having only a middle school diploma. Concerning social media usage, 27.2% of parents had one social media account, 67.2% had two social media accounts, and 5.7% had no accounts (Table 1). Variables associated with the phenomenon of sharenting are reported in Table 2. Approximately two-thirds of the participating parents (65.7%) reported sharing photos of their children on social media platforms, with the frequency of sharing ranging from a few times a year to several times a month or week.

**Table 1:** Profile of the parent sample participating in the study

Characteristics of respondents (100%)	Sex	Female (81.7%) Male (18.3%)
	Age (years)	18–25 (2%) 26–35 (26.4%) 36–45 (25.9%) > 45 (45.7%)
	Education Level	University degree (45.4%) High school diploma (43.7%) Middle school diploma (10.9%)
	Social Media Usage	One social media account (27.2%) Two social media accounts (67.2%) No accounts (5.7%)

*Demographic Profile of Parent Respondents in the Study*

**Table 2:** Behavioral variables associated with the phenomenon of sharenting

Variables		*N* (%)	Sharenting is child neglect and abuse	Sharenting is not a case of child neglect and abuse	χ2 ^_^
How often do you share photos and videos of your children on social media?	Never	139 (34,3%)	123 (88,5%)	16 (11,5%)	29.839 *
	Weekly	25 (6,2%)	14 (44%)	11 (56%)	
	Several times a month	63 (15,6%)	27 (42,9%)	36 (57,1%)	
	A few times a year	178 (44%)	54 (30,3%)	124 (69,7%)	
When did you start sharing photos and videos of your children on social media?	Never	149 (29,8%)	132 (42,1%)	17 (39,7%)	29,720 **
	During pregnancy	12 (6,9%)	16 (4%)	12 (42,9%)	
	At the time of birth	51 (12,6%)	33 (8,1%)	18 (4,4%)	
	Before the age of two	94 (23,2%)	59 (14,6%)	35 (37,2%)	
	After the age of two	83 (20,5%)	57 (14,1%)	26 (31,3%)	

Behavioral Variables of the Parents Participating in the Study. *expected minimum count is 6.67 **. The minimum expected count is 7.47

The content shared often revolved around significant events such as birth or birthdays (50.5%) or the child’s artistic activities (27.3%). The primary reason cited by respondents for sharing content about their children was to create a memory archive (47.5%), followed by making connections (14.6%), informing others (4%), affirming their role as parents (2%), and gaining visibility (1%). Participants expressed concerns about privacy (71.4%) as the aspect most susceptible to harm due to sharing photos and videos on social media. Other perceived harms included emotional harm (12.6%) from potentially hurtful comments and economic exploitation (advertising or profit purposes). Some participants (10.6%) did not report any harmful aspects (Table 3). Participants indicated that mothers and fathers in their households used social media equally (45.6%) and believed that parents should seek permission from their children before sharing information about them (70.6%). Furthermore, 73.3% of participants believed that excessive sharing of children’s photos and videos on social media could be considered child neglect and abuse. The chi-square independence test showed that gender (x 2 = 1,178, *p* > 0,05), age (x 2 = 0,110, *p* > 0,05), education level (x 2 = 0,873, *p* > 0,05), number of children (x 2 = 0,661a, *p* > 0,05), number of social media accounts (x 2 = 4,027, *p* > 0,05) were not associated with sharenting phenomenon. However, the frequency of sharing photos and videos of children and the timing of starting to share were associated with sharenting phenomenon. Those who never shared content related to their children, or only did so a few times a year, were more likely to view excessive sharing as a form of child abuse. The chi-square independence test showed no association between gender, age, education level, number of children, or number of social media accounts and sharenting phenomenon. However, the frequency of sharing photos and videos of children and the timing of starting to share were associated with sharenting phenomenon. Binary logistic regression analysis revealed that frequent sharing of content related to children on social media was a risk factor for not perceiving sharenting phenomenon as neglect and abuse. Conversely, never sharing or sharing infrequently was protective against this perception.

**Table 3:** Perceived risks of harm from sharing visual content of their children among study participants

Type of harm perceived by responders (100%)	Invasion of privacy (71.4%) Emotional harm (12.6%) No harm perceived (10.6%)

*Types of harm perceived by responders in the study from sharing visual material of their children*

## Discussion

Several definitions of sharenting have emerged in recent years. Bezakova et al. define it as the excessive use of social media by parents or guardians to share photos or various home videos of minors with the virtual community, while Brosch et al. describe it as the practice of a parent regularly using social media to communicate a large amount of detailed information about their child [9, 14]. The creation of a child’s digital identity may begin when parents share information about their unborn or newborn child on social media, including shared pictures and events, with or without the child’s consent [15].

In this study, a high percentage of sharenting was reported (65.7% of parents), with rates decreasing from a few times a year (44%) to several times a month (15.6%) or week (6.1%), consistent with findings in the literature. Wagner et al. documented that 40% of parents shared pictures of their children on social media, while Bartholomew et al. found that 98% of mothers and 89% of fathers uploaded photos of their child to Facebook [16, 17]. It was surprising that around half of the parents included in the study were over the age of 45, as a higher percentage was expected in a younger sample raised during the rise of social media, based on previous studies [18]. Millennial parents, born between 1980 and 2000, are considered digital natives. As they become parents, they raise their children in a digital media culture and may be encouraged to record and share activities digitally, viewing sharenting as a social norm in this digital age [18–20]. The older age of the parents in this study, compared to those in other studies, suggests that the phenomenon of sharenting is not limited to parents born in the digital age but spans a broader demographic. This indicates that sharenting may be used as a means of self-gratification and self-representation, regardless of age. Future social studies should investigate the underlying reasons for sharenting and explore the emotional and psychological motivations driving parents to engage in this practice, as well as whether differences exist based on age.

The timing of the onset of sharenting was a focus of this study, which found that 23.2% of parents started sharenting after birth, 20.5% before the age of two, 12.6% at birth, and 6.9% while still in the womb. This is consistent with the results of the AVG survey conducted in several countries, where 81% of children under the age of 2 already had a digital footprint from their parents [4]. The increasingly early creation of digital identities, often without the consent of the children involved, highlights the growing prevalence of sharenting, which is increasingly seen as a normal and risk-free practice. The sharing of images and information about unborn or newborn children suggests that most parents do not perceive this practice as a violation of their children’s privacy or right to self-determination. Most studies reported that parents generally shared happy moments, often recorded during everyday activities, outings, and special events [16]. Brosch et al. found that many parents shared posts with pictures of their children’s birthday parties, baby videos, birth certificates, kindergarten diplomas, or art or sonogram pictures [21]. They also sometimes shared embarrassing photos (e.g., nude or semi-nude pictures of the child bathing or at the beach), photos where children were distressed (e.g., crying or angry), or photos of children covered in food (e.g., chocolate on their faces after dinner) [21]. Conversely, Hashim et al. found that parents mostly shared social events (e.g., vacations, events, family activities, and outings, 29.3%), moments (e.g., good, funny, happy, important, or special moments, 25.3%), day-to-day activities (13.3%), memories of their children (12%), school activities (10.6%), food (4%), antics (2.6%), and milestones (2.6%), while Marasli et al. found that the most common themes parents shared on Facebook were special days (81.4%), such as birthdays, graduations, and year-end shows, followed by social activities (54.98%) and educational issues (30%) [22, 23]. A similar study conducted by Er et al. during the COVID-19 pandemic found that many posts focused on the joy of spending time with children and love toward family, showing children happily playing games, cooking, or learning together. A smaller proportion of posts expressed unpleasant situations, such as boredom and complaints about quarantine [24]. In this study, sharenting content was often related to specific days, such as birthdays (50.5%) or children’s artistic activities (27.3%). It is no coincidence that joyful moments are the ones most frequently posted and shared by parents, as this practice often arises from a desire for self-gratification and the need to present themselves as good parents. In contrast, unpleasant moments, such as those involving sadness or boredom, are typically not shared.

Parents’ motivations for creating social digital identities for their children on social networking sites were explored. For 47.5% of the sample, the main reason for sharing content about their children on social media was to build an archive of memories. For 14.6%, it was to make connections, 4% shared to inform and advise others, 2% to affirm their role as parents, and 1% for visibility.

Our results suggest that parents may engage in sharenting not solely for personal reasons but also to create an easily accessible archive of memories. However, it is likely that parents are either reluctant or embarrassed to reveal their true motivations, especially given that there are alternative methods for preserving memories that are less public and potentially safer.

On the other hand, Briazu et al. found that making social connections and seeking parenting support were primary motivations for mothers, while Fox and Hoy showed that sharenting was often used to project the image of being a good parent, particularly by portraying the child in a favorable light and avoiding posts that could be embarrassing or make the parent appear inadequate [25, 26]. Saving memories was also a common motivation, as noted by Hashim et al., who highlighted that social networks were used as archives of experiences, activities, and feelings that children could recover when they grow up [22].

Data from this study indicate that gender, age, education level, number of children, and number of social media accounts were not associated with sharenting behavior. However, variables such as the frequency of sharing photos and videos of children on social media and the timing of initiating such sharing were found to be linked to sharenting. These findings do not align with those of Balaban et al., where parental gender, age, and sharing status were related to sharenting, suggesting that certain demographic factors may indeed influence the behavior [27].

Lastly, this study’s data suggest that the frequency and onset time of sharenting were the main variables influencing the perception of sharenting as neglect and abuse. When asked whether excessive sharing of children’s photos and videos on social media could be considered child neglect or abuse, 73.3% of participants responded affirmatively. Parents expressed concerns that sharing about their children on social networks could have harmful effects: 71.4% indicated that it could compromise a child’s privacy, leading to neglect and abuse, and that their images could be used on inappropriate sites, while 12.6% pointed to emotional consequences (due to harmful comments) and economic ones (children being exploited for advertising). Only 10.6% reported no adverse effects.

Despite being aware of the potential harm caused by sharenting, the parents in our study continue to engage in this practice, underscoring the need for educational interventions that are currently lacking. Sharenting should be considered a form of child abuse, as it violates children’s privacy and image, especially when content is posted without their consent [1]. Moreover, it exposes children to risks such as sexual exploitation, emotional harm, unlawful access to metadata, and digital kidnapping [1]. For example, many innocent photos of children shared on social media can reappear on pornographic platforms [1].

Sharenting can also affect the psychosocial well-being of children by impacting their self-esteem, autonomy, and self-perception, with potential long-term consequences for their future relationships with both family members and others [28]. Kumar and Schoenebeck introduced the term “privacy stewardship,” describing the responsibility mothers take on when considering the appropriateness of sharing baby photos and their implications for the child’s digital footprint [29]. Cino and Dalledonne Vandini also described the pressures of motherhood, as mothers are expected to actively manage their children’s digital presence [30].

Child privacy is closely linked to digital kidnapping, where people steal a child’s identity and photo on social media, presenting the child as their own [31, 32]. This is one of the risks associated with creating digital identities for children through image sharing, especially when images contain personal information and show the child’s face [24, 31, 32]. However, not all parents seem fully aware of these risks. The Italian study by Cino et al. found that while mothers generally intend to respect their children’s privacy, these intentions often conflict with their desire to share images for various reasons. Although the risks of early internet exposure are widely recognized, there remains a prevailing belief that such actions fall within the “unquestionable” rights of the parent, often overlooking the dangers associated with the virtual world [32]. Therefore, it is crucial to provide parents with informed guidance on whether to share their children’s content online.

Williams-Ceci et al. proposed behavioral interventions aimed at changing parents’ attitudes toward posting information, photos, and videos of their children online by raising awareness of the dangers of sharenting. They tested a video-based intervention that highlighted the permanence of online information and how children might feel about it. The study showed that educational videos reduced parents’ willingness to post content about their children [33]. However, to date, no specific and widely validated interventions have been found to effectively modulate parents’ attitudes toward sharenting. Existing policies addressing sharenting are considered insufficient [1], and it is recommended that these policies be updated to account for online identities. Finally, it is advised to conduct more research on parents’ attitudes toward privacy and the factors influencing their sharing of children’s photos and information online [1, 13].

## Limitations

This pilot study has several limitations. Our Italian sample is not representative of Italian families due to the high proportion of parents with college degrees, which can be attributed to the recruitment method and the motivation to participate. To involve a more diverse population of parents, alternative recruitment channels, particularly non-digital ones, should be considered. This approach could help in recruiting a more representative sample of Italian parents. While the results may be relevant to other social and cultural backgrounds, generalizations to contexts different from Italy should be made cautiously.

Sharenting is a relatively new phenomenon, and the existing literature on this topic is not yet comprehensive, especially regarding large sample sizes. Further research, including comparative studies with larger sample sizes, is necessary to deepen our understanding of the sharenting phenomenon. Comparisons with other surveys may be challenging due to differences in recruitment methods, cultural contexts, and the diverse platforms used (such as Facebook and Instagram). Wherever possible, efforts should be made to minimize these differences and use the same validated questionnaires.

Additionally, it is important to discuss the definition of child abuse with parents before investigating their perceptions. Finally, expanding the study by involving European pediatric societies will be crucial to obtaining comparable data and developing family education programs aimed at limiting the phenomenon of sharenting.

## Conclusion

The phenomenon of parents sharing sensitive content about their children on internet platforms, known as sharenting, is well-established and increasing. Many parents view sharenting as a convenient way to store memories and reminisce about their children’s events and achievements. However, despite its perceived benefits, sharenting exposes children to significant risks and may negatively impact their psychological and physical development and safety, including risks like online harassment, identity theft, and cyberbullying. While traditional forms of child abuse involve direct harm to the child, sharenting can damage the child’s image and pose long-term risks to their well-being, both now and in the future [13].

Sharenting may result in emotional neglect or abuse, as caregivers may prioritize creating content for their online followers over the child’s needs. In some cases, the caregiver may even coerce the child into repeating certain behaviors or phrases for the camera, exploiting them for entertainment purposes. Therefore, sharenting can be considered a form of neglect and abuse, as it endangers the child during the recording process and leaves them vulnerable to future harm from the shared content.

Educational programs and awareness campaigns are urgently needed to address the risks associated with sharenting. Pediatricians can play a vital role in educating parents about the dangers of oversharing online, and specific training for pediatricians could equip them with strategies to address this issue effectively. Families should be encouraged to adopt protective strategies, such as photo editing and self-censorship, to safeguard their children’s privacy. Additionally, parents can be guided to find a balance between sharing pride in their children’s achievements and understanding the associated risks. Offering prenatal courses on social media use and digital literacy could further enhance parents’ awareness.

Preventive interventions, such as counseling and parental education, are essential to protect children’s well-being, privacy, and identity. While the data from this study are limited, they highlight the importance of expanding data collection across different countries. Establishing a comprehensive database could help local and European governments develop policies and educational programs to mitigate the risks associated with the misuse of personal data, ultimately protecting children in both the short and long term.

Validated questionnaires should be used to measure instances of child maltreatment related to sharenting and identify at-risk parents for targeted preventive interventions. Currently, no scientific literature provides evidence of effective interventions to change this practice among parents. Therefore, future research should focus on developing targeted prevention strategies to address this growing concern.

## Data Availability

The datasets used and/or analysed during the current study are available from the corresponding author on reasonable request.
